# Water Transport and Ion Diffusion Investigation of an Amphotericin B-Based Channel Applied to Forward Osmosis: A Simulation Study

**DOI:** 10.3390/membranes11090646

**Published:** 2021-08-24

**Authors:** Hao-Chen Wu, Tomohisa Yoshioka, Keizo Nakagawa, Takuji Shintani, Hideto Matsuyama

**Affiliations:** 1 Research Center for Membrane and Film Technology, Kobe University, 1-1 Rokkodai, Nada, Kobe 657-8501, Japan; waynewu18@shark.kobe-u.ac.jp (H.-C.W.); k.nakagawa@port.kobe-u.ac.jp (K.N.); shintani@port.kobe-u.ac.jp (T.S.); matuyama@kobe-u.ac.jp (H.M.); 2 Department of Chemical Science and Engineering, Kobe University, 1-1 Rokkodai, Nada, Kobe 657-8501, Japan; 3 Graduate School of Science, Technology, and Innovation, Kobe University, 1-1 Rokkodai, Nada, Kobe 657-8501, Japan

**Keywords:** Amphotericin B channel, molecular dynamics, forward osmosis, water transport, ion diffusion

## Abstract

The use of an Amphotericin B_Ergosterol (AmBEr) channel as an artificial water channel in forward osmosis filtration (FO) was studied via molecular dynamics (MD) simulation. Three channel models were constructed: a common AmBEr channel and two modified C3deOAmB_Ergosterol (C3deOAmBEr) channels with different diameters (12 Å and 18 Å). During FO filtration simulation, the osmotic pressure of salt-water was a driving force for water permeation. We examined the effect of the modified C3deOAmBEr channel on the water transport performance. By tracing the change of the number of water molecules along with simulation time in the saltwater region, the water permeability of the channel models could be calculated. A higher water permeability was observed for a modified C3deOAmBEr channel, and there was no ion permeation during the entire simulation period. The hydrated ions and water molecules were placed into the channel to explore the ion leakage behavior of the channels. The mean squared displacement (MSD) of ions and water molecules was obtained to study the ion leakage performance. The Amphotericin B-based channels showed excellent selectivity of water molecules against ions. The results obtained on an atomistic scale could assist in determining the properties and the optimal filtration applications for Amphotericin B-based channels.

## 1. Introduction

Access to potable water is becoming an important issue with the growth of pollution and expansion of the world’s population. In recent decades, water treatment via membrane processing has attracted much interest because of dependable performance and superior efficiency. Due to a shortage of drinking water supply, higher-performing water treatment membranes will be necessary in the near future. In order to cope with the severe demand, membranes with superior flux, high levels of rejection for solutes, and low levels of energy consumption are needed for separation processes. In a positive development, a material referred to as Aquaporin has shown high potential to attain the type of ideal water treatment membrane that will be required [1]. Aquaporin is a type of protein channel that was discovered in the human kidney [2]. In pioneering studies, Aquaporin garnered attention for both rapid water transport and complete ion rejection. This admirable performance has resulted in the introduction of biomimetic membranes. Kumar et al. have successfully prepared a membrane using Aquaporin as a water transport channel with outstanding water permeability [3]. Further, some researchers have claimed that Aquaporin-inspired membranes have exhibited water permeability that is much higher than that of general polymeric RO membranes [1,4]. Although there are numerous advantages in using Aquaporin for water treatment, there also are conspicuous drawbacks. The price and the complex molecular structure of Aquaporin-inspired membranes make them difficult to produce on an industrial scale. Likewise, Gramicidin A (GA) has also been considered a water channel candidate. The simple peptide structure of GA forms a nano-scale channel within a lipid bilayer for water transport. In RO applications, GA has shown performance that approximates that of Aquaporin [5,6], but the toxicity of GA has significantly limited its application. Therefore, the realization of a low-priced, reliable, non-toxic, and safe water channel with high water transport performance has been a vexing pursuit.

The concept of an artificial water channel has been suggested, and proposals have included the macrocycle 4 [7,8,9], the I-quartet [10,11,12], and carbon nanotubes [13,14,15,16]. These all have the potential to form a nano-scale channel within a lipid bilayer that could be developed as a biomimetic membrane. These types of biomimetic membranes are expected to provide water transport performance that is similar to that of Aquaporin, but at a lower cost. Based on the referenced pioneering studies, biomimetic membranes with artificial water channels are expected to be the next generation of membranes because of their unique transport and rejection abilities [7,10,13,17]. Furthermore, the connected-channel design of the structure could be suitable for a forward osmosis process [18,19,20,21,22], which is difficult to achieve with general polymeric membranes. In the present study, Amphotericin B-Ergosterol (AmBEr) was examined as another potential candidate for use as an artificial water channel. An AmBEr Channel is a type of self-assembly channel composed of Amphotericin B and Ergosterol monomers, which can be embedded into a lipid bilayer to form a connected channel structure for a bio-inspired membrane with high biocompatibility [17,23,24].

In order to develop an energy-saving biomimetic water channel membrane with a high degree of water flux and high solute leakage, a simulation technique was used to examine the characteristics of materials on an atomistic scale. The benefit of a microscopic viewpoint is a deeper understanding of the structural properties and transport mechanisms of artificial water channels, which is paramount for improving material development. Previous productive research achievements via simulation techniques have promoted experimental work. In the case of a natural water channel such as the Aquaporin channel, transport properties such as permeability and selectivity have been deeply discussed [25,26,27,28,29]. In addition, the features of a GA channel have been widely studied via Monte Carlo and molecular dynamics simulation techniques [30,31,32,33,34]. In previous attempts to realize an artificial water channel, three types of materials were studied. First, as a candidate for an artificial water channel, carbon nanotubes have been broadly explored via theoretical methods [35,36,37,38,39,40]. Second, a kind of self-assembled channel, the cyclic peptide nanotube (CPNT), was thoroughly examined in our previous studies. Four types of CPNTs were studied to compare structural features and water permeation performances [41]. Additionally, the application of CPNTs to the FO process was introduced in our previous study [42]. The above studies have supplied plenty of advantageous data, which would have been difficult to obtain from actual experimental work. Another key point to remember is that the results from simulation studies have shown tendencies similar to the experimental results. Theoretical calculation on a molecular level is considered a feasible tool for the development of this type of novel membrane [41,42,43,44,45]. The present study in molecular dynamics simulation introduces Amphotericin B–Ergosterol (AmBEr) artificial water channels that are applicable to FO filtration. Two types of AmBEr channels were constructed for FO filtration simulation. First, the water molecular transport performance was studied within a FO simulation model. Second, how chemical modification and channel size affected the ion leakage ability of the channel was also explored via simulation. A hydrated ion structure composed of ion atoms and water molecules was placed directly into the channel model to investigate the ion leakage mechanisms. Relative mobility was adopted to investigate the selectivity between ion atoms and water molecules via mean square displacement (MSD) analysis.

## 2. Simulation Method

In order to better understand the practical applications and ion leakage mechanisms of an Amphotericin B-based channel, a molecular simulation model of forward osmosis (FO) was introduced in this work. Two types of channels in three simulation models, AmBEr and C3deOAmBEr with different channel diameters, *d* (*d* = 12 Å and *d* = 18 Å), were constructed to study the FO process on an atom scale. All these molecular models were built using BIOVIA Materials Studio commercial software (San Diego, CA, USA).

### 2.1. Molecular Model of an Amphotericin B-Ergosterol Channel

**Figure 1:**
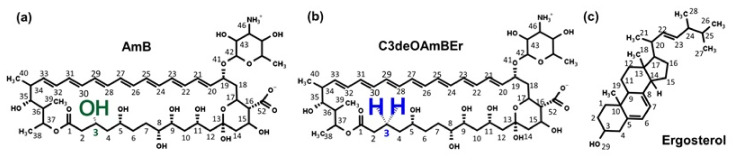
Chemical structures of simulation material monomers; (**a**) Amphotericin B (AmB), (**b**) modified Amphotericin B (C3deOAmBEr) and (**c**) Ergosterol monomer.

In our studies [43,45], an Amphotericin B-based channel has been studied for use as an artificial water channel in water treatment. In the present study, specific hydrogen atoms within the Amphotericin B monomers were modified to enhance water permeance. A common AmBEr channel model with an initial diameter of 12 Å and two C3deOAmBEr models with different initial diameters of 12 and 18 Å were constructed. The chemical structures of objective material monomers appear in Figure 1. In the C3deOAmBEr model, a hydroxyl group located at the #3 carbon was replaced by a hydrogen atom to create a more hydrophobic channel surface.

All simulation models were constructed from 16 Amphotericin B and 16 Ergosterol monomers using the Amorphous Cell module of the Materials Studio, then adopting the “Geometry Optimization” function in the energy-optimizing step. A Condensed Phase Optimized Molecular Potential for Atomistic Simulation Studies (COMPASS) [46,47,48] force field was selected to perform the energy minimization and MD calculation processes. Details of the model construction procedure and the force field are included in the supplementary information.

### 2.2. Molecular Model of Forward Osmosis Simulation

**Figure 2:**
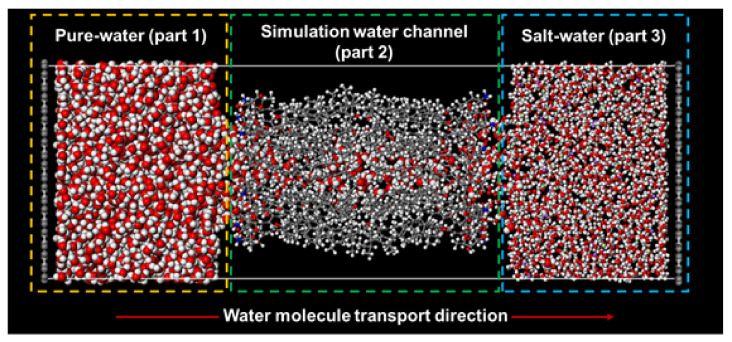
Forward osmosis filtration simulation model.

The forward osmosis simulation model appears in Figure 2. The simulation model consisted of three parts. From left to right in the figure, the pure water receptacle was located in part 1. A channel filled with water molecules was located in the center (part 2), and a saltwater receptacle was located in part 3. To simulate FO, we used an aqueous salt solution of Na^+^, Cl^−^ ion atoms and water molecules, which corresponded to a molarity concentration of about 1.70 M. Details of the FO simulation models are listed in Table 1. The FO system is different from a real application system such as actual seawater desalination. The purpose of this paper is to examine the potential of membrane performance of AmBEr water channels in FO mode. Therefore, we modeled the FO system consisting of the simplest NaCl aq.-Pure water system. In practical applications, an aqueous solution to be dehydrated should be placed on the feed side, and a draw solution with a higher osmotic pressure than the aqueous solution in feed should be placed on the permeate side.

As shown in Figure 2, the periodic boundary conditions in the z direction were cut by two fixed graphene plates. These graphene plates served as a barrier to shut off interaction between the pure-water and saltwater receptacles [49]. Normally, this type of bio-inspired channel-based membrane uses channel materials encircled by a lipid bilayer to maintain the membrane structure, which was too heavy for computer simulation. To solve this problem, we referred to previous studies [43] where the movement of the carbon atoms of Amphotericin B monomers could be constrained and fixed at specific positions to maintain the channel structure, instead of setting an ambient lipid bilayer. In order to maintain flexibility in the simulation channel model, the number of constrained carbon atoms was limited to around 5% of the total number of atoms. Although this simplified method could have led to slight distortions when mimicking the osmotic pressure-driven FO filtration process, the effect on the transport and leakage mechanisms of these three simulation channel models was insignificant [50].

### 2.3. Physical Property Analysis

The water-molecular transport performance and ion-leakage capability of two types of Amphotericin B-based channels was studied using an FO simulation model and a simulation technique. Details of the physical properties are described in this section.

#### 2.3.1. Hydrated Structure of Ions

In the present work, we aimed to mimic the atom-scale Amphotericin B-based channel transport performance via an osmotic pressure-driven FO filtration simulation model. Hence, validation of the hydrated structure of ions and the feasibility of a force field was necessary. The hydrated structure of Na^+^ and Cl^−^ ions was scrutinized via radial distribution function (RDF) in our previous study [42]. Further, there was good agreement between the hydrated diameter, the simulation results, and the experimental data in certifying the feasibility of the COMPASS force field [42].

#### 2.3.2. Prediction of Water Permeability

The transport behavior of water molecules in the simulation models was observed via MD simulation. In the calculation period, the number of water molecules was tracked and recorded in each of the parts. In other words, the time-course curves for the number of water molecules as a function of time within the channel simulation model were obtained via the NVT MD simulations. Alterations of the water molecules were observed to analyze the permeation behaviors of the simulation system. The osmotic pressure is considered the driving force of the FO process, and in this work the osmotic pressure was calculated using the Van’t Hoff equation. Then, in order to predict the water permeability, the slope of the curve for water molecules was adopted and used to calculate the permeability via Equation (1).

(1) $$permeability\;\left[mol\;m/(m^{2}s\;Pa\right)]=\frac{\Delta N\;L}{A\;\Delta t\;\Delta \pi N_{A}}$$

In Equation (1), Δ*N* [–] is the change in the number of molecules during a certain period of time, Δ*t* [s], *A* [m^2^] is the area of the cross-section of the Amphotericin B-based channel, Δπ is the mean osmotic pressure during Δ*t*, *N*_A_ [mol*^−^*^1^] is Avogadro’s number, and *L* [m] is the channel length, where *L* = 5.2 × 10*^−^*^9^ m. In the present study, water molecules were inserted into the simulation channel in its initial state as a hydrated water channel, and a 10 ns calculation period provided diagrams for the time course of the number of water molecules. The details of the calculation procedure appear in the supplementary information.

#### 2.3.3. Ion Leakage Capability

The ion rejection/leakage ability in a saltwater treatment process is an important feature for an RO or FO membrane, and attention should always be paid not only to water permeability but also to ion rejection/leakage by the membrane. In the present work, the permeation of ions was traced through the channels. The saltwater receptacle consisted of sodium ions, chlorine ions, and water molecules. The initial ion concentration as a molarity of the saltwater receptacle was set to approximately 1.70 M. During the FO simulation period, the transport behavior of ions through the channel was traced and observed. However, no ions permeated any of the Amphotericin B-based channels, which indicated a high potential for ion leakage by these channel models. Therefore, to investigate further how the modified AmBEr channel affected the ion leakage ability, hydrated ions were released into the channel model, and their diffusion behavior was directly traced. Values for displacement and trajectory were recorded as MSD. Through analysis of the relative MSD between water molecules and ion atoms, the ion leakage performance for each channel model could be discussed. The mean square displacement, MSD, defined by Equation (2), was calculated for the water molecules, Na^+^ ions, and Cl^−^ ions inside the AmBEr-based channels.

(2) $$MSD(t)=\frac{1}{N}\sum_{i=1}^{N}\langle {\left[r_{i}(t_{0}+t)-r_{i}(t_{0})\right]}^{2}\rangle$$

The diffusion coefficient, D, of these species was estimated using Einstein’s relationship between MSD and D given by Equation (3), where B is a constant, t is time, and dn is the dimension of the diffusion space, which is normally 6 for free diffusion in a 3-dimensional bulk space. For the case of 1-dimensional diffusion through a straight channel, 2 is adopted as a value of dn [43].

(3) $$B+d_{n}Dt=MSD(t)$$

## 3. Results and Discussion

### 3.1. Water Permeability

In the FO filtration simulation process, water molecules were transported from a pure-water receptacle to a saltwater receptacle. We observed the transport behavior as it was driven by osmotic pressure within different simulation channel models, which allowed study and comparisons of the leakage mechanisms.

#### 3.1.1. Common AmBEr Channel (*d* = 12 Å)

**Figure 3:**
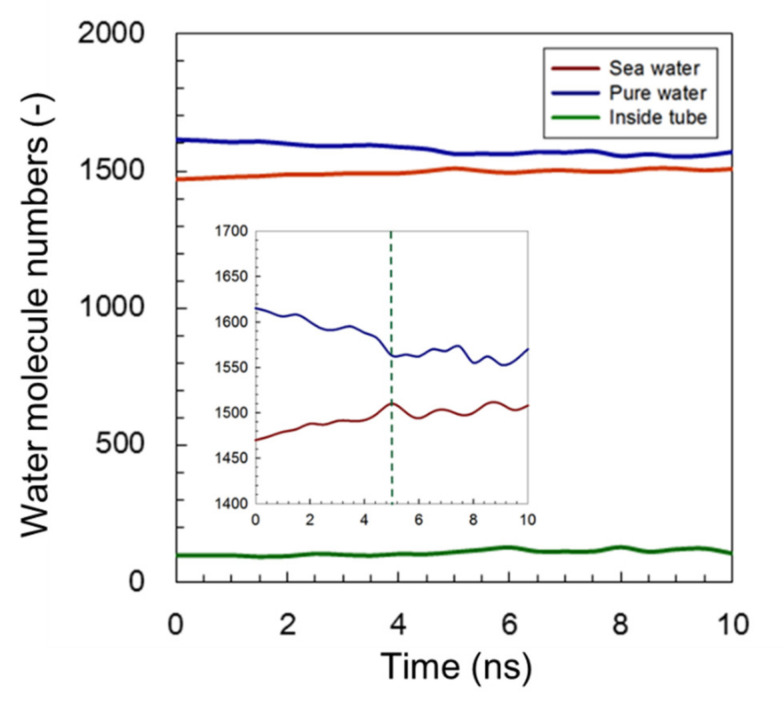
Time course for the number of water molecules during FO simulation when using a common AmBEr channel (*d* = 12 Å). Inserted figure shows the enlarged vertical axis.

As shown in Figure 3, once the transport process was started, the amount of water molecules in the pure-water receptacle was decreased and the amount within the saltwater receptacle was increased until the simulation system reached a state of equilibrium after approximately 5 ns. The initial amount of water molecules in the simulation channel remained consistent. An enlarged vertical axis rapidly altered the number of water molecules just after the simulation had started, and the slope then gradually became stable until a state of equilibrium was reached at a time of approximately 5 ns. Time course of water molecule number during FO process simulation is depicted in Figure S1. This tendency resulted in a slope between the pure water and the saltwater that was roughly approximate. The occurrence of this similar slope alteration reflected the adequacy of this FO simulation system. Based on the slope of the time-course curve, the water permeance was predicted to be 3.3 × 10^−5^ (mole s^−1^ m^−2^ Pa^−1^) per channel within a range of from 0 to 5 ns, and by considering the length of the channel, *L* = 5.2 × 10^−9^ m, the corresponding value of water permeability was evaluated as 1.7 × 10^−11^ (mole m s^−1^ m^−2^ Pa^−1^). 

With respect to novel Amphotericin B-based material, if this channel could be embedded into a lipid bilayer to form a biomimetic membrane with a porosity of 1%, the water permeance of a common AmBEr channel could be calculated to be around 3.3 × 10^−5^ (mole s^−1^ m^−2^ Pa^−1^). For validation, the experimentally estimated water flux of the AmBEr channel-derived membrane has been reported as approximately 6.7 × 10^−5^–3.7 × 10^−4^ (mole s^−1^ m^−2^ Pa^−1^) [51]. The resultant good agreement between the simulated permeance and the experimental data suggests that this simulation technique could be an asset to experimental work in the design of a novel material. 

#### 3.1.2. Modified C3deOAmBEr Channel (*d* = 12 Å)

**Figure 4:**
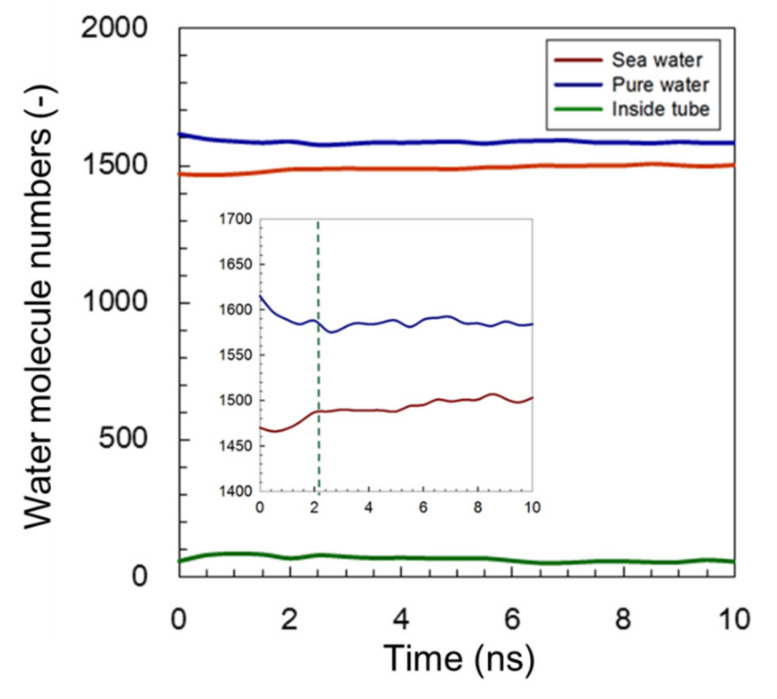
Time course of the number of water molecules during FO simulation using a modified C3deOAmBEr channel (*d* = 12 Å). Inserted figure shows the enlarged vertical axis.

A similar tendency for variation in the number of water molecules was also observed in the modified C3deOAmBEr channel model, as shown in Figure 4. However, compared with the results using a common AmBEr channel with the same diameter, *d* = 12 Å shown in Figure 3, a somewhat shorter variation period was seen for the modified C3deOAmB (*d* = 12 Å) channel shown in the inserted enlarged figure in Figure 4. As mentioned in our previous study [43], the C3deOAmB channel became more hydrophobic when modified by hydrogen atoms. This modification to enhance the hydrophobicity at the middle of the channel was considered one of the methods that could be used to improve the ability of reject ion/leakage [52]. In order to compare the water permeation performance of two different simulation models, the same calculation method was followed, and the slope was adopted to predict the value of the water permeability. In the case of the C3deOAmB channel, a steady state was observed at a simulation time of around 2 ns. Therefore, a mean slope of 3 ns was adopted, and the value of water permeability was calculated at approximately 2.2 × 10^−11^ (mole m s^−1^ m^−2^ Pa^−1^).

The value of water permeability for the modified C3deOAmBEr (*d* = 12 Å) was somewhat higher than that for a common AmBEr channel. In our previous study [43], the diffusion behavior of water molecules under an adsorption equilibrium state was discussed to predict water permeability via a solution-diffusion model, which illustrated how modification that enhances the hydrophobicity simultaneously, and dramatically, increases the water transport and ion leakage performance. In the case of the C3deOAmBEr (*d* = 12 Å) model, although the affinity turned more hydrophobic and showed higher performance in permeability, the low-affinity to water molecules and small inner diameter would be effective for a quick stabilization of the system. The C3deOAmBEr system more quickly reached an equilibrated steady state where osmotic pressure and density of the saltwater receptacle was balanced, which could have led to the short alteration period for the number of water molecules.

#### 3.1.3. Modified C3deOAmBEr Channel (*d* = 18 Å)

**Figure 5:**
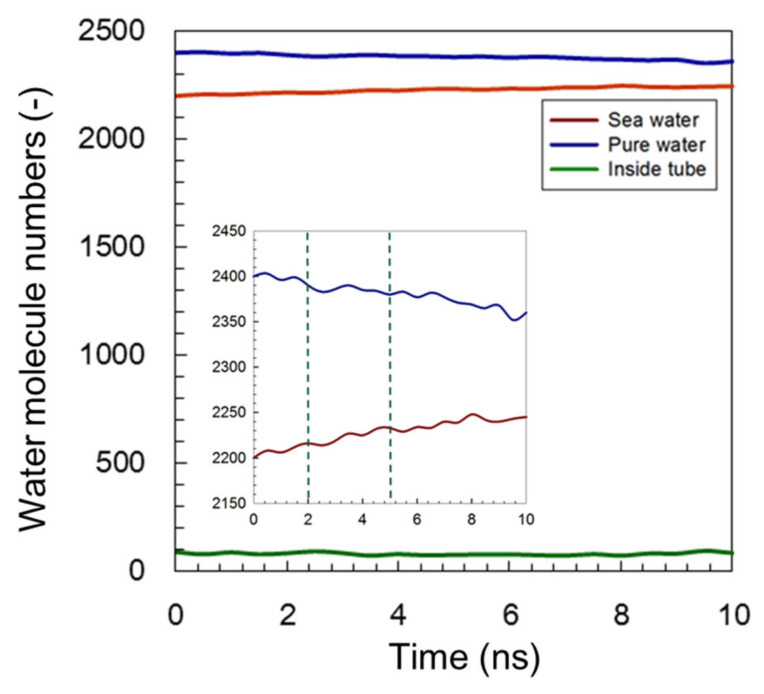
Time course for the number of water molecules during FO simulation with a modified C3deOAmBEr channel (*d* = 18 Å). Inserted figure shows the enlarged vertical axis.

The C3deOAmBEr (*d* = 18 Å) model was the most stable modified channel model [43] with a unique permeability performance that altered the number of water molecules, as shown in Figure 5. Although the C3deOAmB (*d* = 18 Å) model had a similar transport mechanism, it also revealed the longest water molecular transport period. Figure 5 shows how the common AmBEr and modified C3deOAmBEr (*d* = 12 Å) models maintained a constant flow of water molecules within the channel, but it showed transport behavior that was driven by the ion concentration difference during the entire simulation period. This long-period water molecular transport was attributed to the larger size of the channel. The water molecules behaved like bulk water affected by its macroscopic viscosity in such a large space, and they could not be driven by the specific acceleration effects for transport. This could have increased the resistance between the pure-water and saltwater receptacles. Although the channel diameter was enlarged, there was no ion permeation observed during the entire simulation period. This result could reflect the low ion leakage ability of the modified C3deOAmBEr channel (*d* = 18 Å) as well as other models with smaller channel diameters.

#### 3.1.4. Overall Comparison of Water Permeability

To study the transport behavior and ion leakage mechanism of an artificial Amphotericin B-based channel, the predictions for the water permeability of three simulation channel models were compared. Because the water transport period was different for each of the simulation models, the water permeability values were predicted from a suitable period, as shown in Table 2. As mentioned in a previous section, the common AmBEr model showed good agreement between the simulation and experimental results, which validated our simulation method. Further, the common AmBEr model could be a standard used to compare modified channel models. The modified C3deOAmBEr channel had a diameter of 12 Å, which was the same as that of the common AmBEr model, but was smaller than that of the C3deOAmBEr (*d* = 18 Å) model. A more hydrophobic nature than that of the common AmBEr would result in higher water permeability, but despite the smaller size of the channel, the permeability of the modified C3deOAmBEr channel was slightly higher than that of the C3deOAmBEr (*d* = 18 Å) model, which unexpectedly showed the lowest permeability of the three models. This result suggests that a channel that is too large negates the water permeability enhancement benefits of a nano space channel. The water permeability shown in Table 2 is normalized per channel cross-sectional area. In terms of permeation, since the contribution of water transport in the central part of the channel, which was far from the channel surface and was not easily affected by its hydrophobicity, was greater than the enhancement of diffusivity by the hydrophobic channel surface, it is considered that the larger channel had a smaller amount of water permeation per cross-sectional area. Another aspect that should be noted is that even with an enlarged channel diameter there was no permeation by ions. 

### 3.2. Ion Transport Behaviors

**Figure 6:**
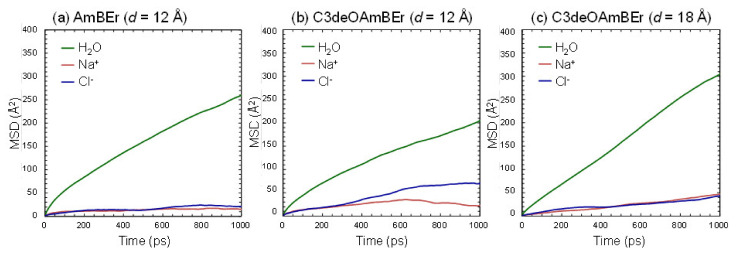
Mean square displacement (MSD) of water molecules and ions within (**a**) the AmBEr channel (*d* = 12 Å), (**b**) the C3deOAmBEr channel (*d* = 12 Å), and (**c**) the C3deOAmBEr channel (*d* = 18 Å).

In the FO permeability prediction section, we found no ion permeation in any of the simulation channel models during the entire simulation period, and this low level of leakage performance piqued our interest. Unfortunately, a long-term simulation period will be necessary to elucidate the ion leakage ability and the selectivity performance of the Amphotericin B-based channels. In order to reduce the computer loading and simulation time, we directly released the fluid composed of ions and water molecules into each channel model over a 1000 ps simulation period. The MSD of the three simulation models was analyzed to examine the ion leakage mechanisms of the Amphotericin B-based channel on an atom scale. The results appear in Figure 6 and Table 3. In all the simulation models, the diffusivity of water molecules inside the channels was lower than that previously reported for pure water [43], although the results showed a relatively higher level of diffusivity for water molecules compared with hydrated ions. In the case of the modified C3deOAmBEr (*d* = 12 Å) model, the difference in the slopes of the MSD curve between water and ions was smaller than that of the common AmBEr model, which indicated a lower level of selective performance. This was due mainly to the lower diffusivity of water molecules in the channel. In a smaller hydrophobic channel, the diffusivity of water molecules would be more effectively reduced by the coexistence of hydrated ions that naturally have a lower level of diffusivity than water molecules. On the other hand, it was interesting that the C3deOAmBEr (*d* = 18 Å) model maintained diffusion selectivity similar to that of the common AmBEr channel model when a large hydrated structure of ions located within the C3deOAmBEr (*d* = 18 Å) simulation model created a level of hydrophobicity in the hydrated structure that was too strong to be easily destroyed. This could also be effective for maintaining a large bulk water cluster [43], which could lead to a lower level of water flux (volume flow rate per cross channel area) in a larger hydrophobic channel.

## 4. Conclusions

Both the feasibility and the channel properties of AmBEr and C3deOAmBEr channels were explored in our previous studies [43,45] through the equilibrated adsorption and diffusion simulations of water molecules. In addition to the previous work, two different points were investigated in the present work. The first point was the application of Amphotericin B-based channel models to FO filtration simulation under non-equilibrium water permeation conditions to examine a more realistic approach to water permeability. The second point involved an examination of the ion leakage capability of the channel models based on comparing the diffusivity of water with that of hydrated ions inside the channels. 

Three Amphotericin B-based channels were osmotic pressure-driven FO simulation models that consisted of a pure-water receptacle and a saltwater (10 wt%) receptacle. These were subjected to a period of 10 ns calculation, which illustrated channel properties that distinguished the common and modified models. The observed water permeabilities were on the order of C3deOAmBEr (*d* = 12 Å) > non-modified AmBEr (*d* = 12 Å) > C3deOAmBEr (*d* = 18 Å). The hydrophobicity of a channel enhanced the mobility of water molecules within it, while a larger size did not [43]. This caused the lower FO water permeability of the C3deOAmBEr (*d* = 18 Å) model despite its higher capacity for water adsorption.

In all three models, diffusion selectivity was higher for water than for salt ions. From the outset, modification of the Amphotericin B monomer resulted in hydrophobicity that not only adjusted the water permeability but also changed the ion leakage ability due to low diffusivity inside the channel. The observed tunable permeability and selectivity reflected the high potential of Amphotericin B-based material for use as an artificial water channel.

In order to achieve a realistic application of biomimetic membranes embedded with Amphotericin B-based material, the properties of this material must be fully elucidated. From that standpoint, novel designs and development of biomimetic membranes relies on further computational simulations and analytical methods on an atomistic-scale. Unfortunately, this paper does not provide details on the atomic-level mechanisms of transport and selectivity. The diffusion mechanism of water molecules at the atomic level in the AmBEr channel is shown in [45], and findings on the effect of charge distribution from functional groups in the channel on the diffusivity of ions in the water channel, although which has a different channel shape from AmBEr, have been reported in [42]. The discussions and detailed study on the permeation/blocking mechanisms from an atomistic scale in the water channel targeted in this paper would be the important subjects in the future work report.

## Figures and Tables

**Table 1:** Details of the forward osmosis simulation model.

	Pure-Water Receptacle No. of Water Molecule	Salt-Water Receptacle No. of Water Molecule	Ion Atoms Na^+/^Cl^−^
AmBEr (*d* = 12 Å)	1615	1470	50/50
C3deOAmBEr (*d* = 12 Å)	1615	1470	50/50
C3deOAmBEr (*d* = 18 Å)	2400	2200	75/75

**Table 2:** Comparison of the calculated water permeability (10^−11^ mole m s^−1^ m^−2^ Pa^−1^) for the three simulation channel models.

	AmBEr (*d* = 12 Å)	C3deOAmBEr (*d* = 12 Å)	C3deOAmBEr (*d* = 18 Å)
0–2000 ps	1.2	2.2	0.4
0–5000 ps	1.7	0.9	0.3
0–10,000 ps	0.7	0.5	0.3

**Table 3:** Calculated diffusivity of water molecules and hydrated ions inside the channels.

	*D*_W_ (10^−9^ m^2^ s^−1^) (Without NaCl)	*D*_W_ (10^−9^ m^2^ s^−1^) (With NaCl)	*D*_Na_^+^ (10^−9^ m^2^ s^−1^)	*D*_Cl_^−^ (10^−9^ m^2^ s^−1^)
AmBEr (*d* = 12 Å)	2.2 [43]	1.12	0.039	0.083
C3deOAmBEr (*d* = 12 Å)	2.4 [43]	0.84	0.061	0.375
C3deOAmBEr (*d* = 18 Å)	3.1 [43]	1.53	0.224	0.155

## Data Availability

Not applicable.

## Supplementary Materials

The following are available online at https://www.mdpi.com/article/10.3390/membranes11090646/s1, Figure S1: Time course of water molecule number during FO process simulation with (a) Common AmBEr (d = 12 Å), (b) Modified C3deOAmBEr (*d* = 12 Å), and (c) Modified C3deOAmBEr (*d* = 18 Å) channel model.
