# Supplementary material for: Water Transport and Ion Diffusion Investigation of an Amphotericin B-Based Channel Applied to Forward Osmosis: A Simulation Study
Source: Membranes (Basel). 2021 Aug 24;11(9):646. doi: 10.3390/membranes11090646 (PMC8467697; doi:10.3390/membranes11090646)
Supplement: Supplementary file 1 [file membranes-11-00646-s001.zip › membranes-1268296-supplementary.pdf]

membranes

MDPI

Supplementary Material

Water Transport and Ion Diffusion Investigation of an Amphotericin B-based Channel Applied to Forward Osmosis: A Simulation Study

Hao-Chen Wu [1,2], Tomohisa Yoshioka [1,3]*, Keizo Nakagawa [1,3], Takuji Shintani [1,3], Hideto Matsuyama [1,2]

1 Research Center for Membrane and Film Technology, Kobe University, 1-1 Rokkodai, Nada, Kobe 657-8501, Japan; waynewu18@shark.kobe-u.ac.jp (H. W.); tom@opal.kobe-u.ac.jp (T. Y.); matuyama@kobe-u.ac.jp (H. M.)

2 Department of Chemical Science and Engineering, Kobe University, 1-1 Rokkodai, Nada, Kobe 657-8501, Japan

2 Graduate School of Science, Technology, and Innovation, Kobe University, 1-1 Rokkodai, Nada, Kobe 657-8501, Japan; k.nakagawa@port.kobe-u.ac.jp (K. N.); shintani@port.kobe-u.ac.jp (T. S.)

* Correspondence: tom@opal.kobe-u.ac.jp; Tel.: +81-78-803-6299

Model construction

BIOVIA Materials Studio® commercial software with a COMPASS force field was used for the design and modeling via molecular simulation [1-3].

At first, Three Amphotericin B-based channels were constructed and their geometries were optimized to obtain the most reasonable initial structures. Secondly, a rectangular-shaped simulation unit cell was prepared and divided into three parts in the construction step of the forward osmosis (FO) simulation model. Periodic boundary conditions (PBC) were employed for the simulation cell, but the PBC was removed only in the *z*-direction by installing two fixed graphene layers at the two ends of the cell for forward osmosis (FO) calculation to prevent mixing the saltwater and pure water. The Amphoericin B-based channel model was located at the center part of the unit cell, and a saltwater receptacle was set at the right-hand side while a pure-water receptacle was located at the left-hand side. After model construction, “Geometry Optimization” was introduced to stabilize the energy of the model structure. In this process, energy calculation and changes in the structures of 5,000 iterations were carried out to establish a sensible initial channel structure.

Finally, a molecular dynamics (MD) simulation of forward osmosis (FO) water transport phenomena was carried out for 10 ns under a canonical (*NVT*, fixed atom number, *N*, system volume, *V*, and temperature, *T*) ensemble at 298 K (at room temperature) to reach an equilibrated state, where the osmotic pressure was balanced against the liquid pressure difference caused by the density difference between saltwater and pure-water.

Prediction of water permeability

The transport phenomenon (alteration of the number of water molecules) was analyzed under a quasi-non-equilibrium state for prediction of water permeability.

To predict water permeability, the number of water molecules within the AmBEr-based channels and in the pure-water, and saltwater reservoirs was traced and recorded. In this work, the driving force of water permeation during the FO simulation period was osmotic pressure, which was calculated using the Van’t Hoff equation ($\pi = iCRT$).

In the Van’t Hoff equation,   [Pa] is the osmotic pressure, C [mol/m^3] is the molarity in the pure water reservoir, R [J/(mol K)] is the ideal gas constant, T [K] is the temperature, and i is a dimensionless correction factor that depends on the degree of the dissociation

of ions. A value of $i = 2$ was reasonably used in this work to evaluate because all Na^+ and Cl^- ions always exist in a saltwater reservoir. The slope of the time course of the number of water molecules was used to calculate water permeability. As shown in Fig. S1 (same as Figs 3(b), 4(b), and 5(b) in the main text), the slope became almost stable after simulation; therefore, this period was adopted for water permeability [mol m / (m² s Pa)] calculation via the following equation. The osmotic pressure was slightly decreased during this period due to an increase in the number of water molecules in the saltwater reservoir. Therefore, we have adopted the mean osmotic pressure during the period as the driving force of water permeation.

$$\mathrm{Permeability}_{\mathrm{H_2O}} = \frac{(\mathrm{slope}\ \left[\frac{1}{\mathrm{s}}\right]) \times (\mathrm{channel\ length\ (thickness)\ [m]})}{(6.02\times10^{23}\ \left[\frac{1}{\mathrm{mol}}\right]) \times (\mathrm{channel\ area\ \ [m^2]}) \times (\mathrm{mean\ osmotic\ pressure\ [Pa]})}$$

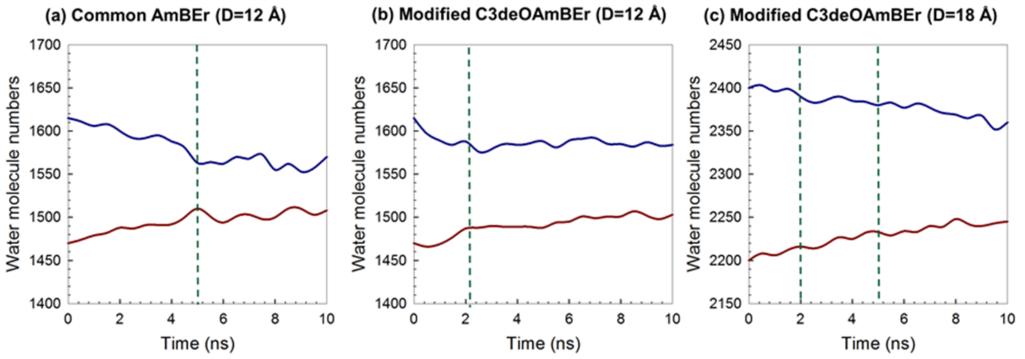

Figure S1. Time course of water molecule number during FO process simulation with (a) Common AmBEr (*d* =12 Å), (b) Modified C3deOAmBEr (*d* = 12 Å), and (c) Modified C3deOAmBEr (*d* =1 8 Å) channel model.

References

[1] H.-C. Wu, T. Yoshioka, K. Nakagawa, T. Shintani, T. Tsuru, D. Saeki, Y.-R. Chen, K.-L. Tung, H. Matsuyama, Water transport and ion rejection investigation for application of cyclic peptide nanotubes to forward osmosis process: A simulation study, Desalination, 424 (2017) 85-94.

[2] H.-C. Wu, T. Yoshioka, K. Nakagawa, T. Shintani, T. Tsuru, D. Saeki, A.R. Shaikh, H. Matsuyama, Preparation of Amphotericin B-Ergosterol structures and molecular simulation of water adsorption and diffusion, Journal of Membrane Science, 545 (2018) 229-239.

[3] H.-C. Wu, T. Yoshioka, K. Nakagawa, T. Shintani, D. Saeki, H. Matsuyama, Molecular simulation of a modified amphotericin B-Ergosterol artificial water channel to evaluate structure and water molecule transport performance, J. Membr. Sci., 583 (2019) 49-58.
